# Game Demands of a Professional Ice Hockey Team with Special Emphasis on Fatigue Development and Playing Position

**DOI:** 10.2478/hukin-2022-000078

**Published:** 2022-11-08

**Authors:** Vincenzo Rago, Adrian Muschinsky, Kasper Deylami, Jeppe F. Vigh-Larsen, Magni Mohr

**Affiliations:** 1 Al Ain Football Club, Al Ain, Emirates Arab United.; 2 Department of Sports Science and Clinical Biomechanics, Faculty of Health Sciences, University of Southern Denmark, Odense, Denmark.; 3 Centre of Health Sciences, Faculty of Health, University of the Faroe Islands, Tórshavn, Faroe Islands.

**Keywords:** match-analysis, testing, physiology, performance, heart rate

## Abstract

The aim of this study was to describe the game activity profile of a professional ice hockey team with special emphasis on fatigue development and playing position. Data were collected using a wearable 200-Hz accelerometric system and heart rate (HR) throughout eight official games in a professional ice hockey team (6 defensemen and 11 forwards; n = 122 files). On-ice 10- and 30-m sprint performance, repeated sprint ability and HR responses to the submaximal Yo-Yo Intermittent recovery level 1 test were assessed to determine associations with game performance. Although the 3^rd^ period was largely longer than the 1^st^ and 2^nd^ periods (r = 0.56–0.59), no differences were observed between periods in activity pattern, except a moderate decline in the number of decelerations <-2 m·s^-2^ per min (Dec2/min) in the 2^nd^ period for forwards (r = 0.06–0.60). Mean HR, time spent >85% HRmax (t85HR), as well as the total number of intense accelerations and decelerations were higher for defensemen. However, demands were similar when expressed relative to time on-ice, except that defenders performed more Dec2/min than forwards in all periods, whereas forwards spent more t85HR during the 2^nd^ period (r = 0.46–0.57). Time spent on ice was inversely correlated with the total number of accelerations (Acc_tot_), accelerations >2 m·s^-2^ per min (Acc2/min), total decelerations per min (Dec_tot_/min), Dec2/min and t85HR (r = -0.63 to -0.18) and positively correlated with mean HR and peak HR (r = 0.20– 0.53). No significant correlations were found between physical fitness and game activity variables scaled by individual time on ice. Absolute acceleration and HR demands of professional ice hockey seem to differ between playing positions, but not in relation to time on ice. Further, no clear signs of fatigue were captured, possibly due to the longer duration of rest intervals in the 3^rd^ period.

## Introduction

Ice hockey is an intermittent team-sport characterized by short duration of exercise (15–25 min), with each shift lasting only 30–80 s, separated by 2–3 min of recovery or longer (Montgomery, 1988). Professional male ice hockey players skate approximately 4–5 km during a game with almost 50% as high-intensity skating, resulting in an average on-ice heart rate (HR) of ~85% maximum HR (HR_max_), confirming both substantial aerobic and anaerobic components (Lignell et al., 2018; Kokinda et al., 2012; Montgomery, 1988; Stanula et al., 2016; Vigh-Larsen et al., 2020a). However, sparse scientific information is currently available regarding the position-specific physical and physiological demands of professional ice hockey match-play, and their relationship with ice hockey-specific fitness profiles.

Match-analysis reports have revealed that activity demands might vary, or not, across playing positions, as well as at different stages of the game as consequence of fatigue development and/or possible tactical alterations (Allard et al., 2020; Brocherie et al., 2018; Lignell et al., 2018; Vigh-Larsen et al., 2020a). Reductions in game performance in male ice hockey have been documented in various studies reporting decreases in average skating speed (Lignell et al., 2018), the amount of efforts with drastic forward lean (Brocherie et al., 2018), total number of accelerations and decelerations (Vigh-Larsen et al., 2020a), accelerations >0.3 m·s^2^ (Allard et al., 2020) and the amount of distance >24 km·h^-1^ covered (Douglas and Kennedy, 2020) during the latter stages of the game, which are supported by a decrease in repeated sprint ability (RSA) after an experimental ice hockey game (Vigh-Larsen et al., 2020a). In contrast, increased skating distance >17 km·h^-1^ covered per minute played during the 3^rd^ game-interval compared to the 1^st^ and the 2^nd^ period has been observed (Lignell et al., 2018) as well as an unchanged activity profile across periods for defensemen (Allard et al., 2020; Stanula et al., 2014, 2016). Previous studies investigating alterations in the work rate during an ice hockey game are limited to three studies examining one single game (Brocherie et al., 2018; Lignell et al., 2018; Vigh-Larsen et al., 2020a), and three studies considering multiple matches (4 to 76 files (Allard et al., 2020; Douglas and Kennedy, 2020; Stanula et al., 2016)). In this context, it is important to consider multiple game datasets to reduce the bias associated to the match-to-match variability in physical performance during official team sports games (Gregson et al., 2010). Additionally, one key aspect of the majority of aforementioned studies is the application of speed-based locomotion variables. In ice hockey, frequent accelerations and decelerations are performed and are likely to contribute significantly to the total match load (Rago et al., 2020, 2021). Moreover, the unique feature of ice hockey being performed on a smooth ice surface with minimal friction allows players to glide across the ice at high speeds with low energy cost after an initial acceleration. Thus, acceleration-based variables should be considered beyond distance-based variables in order to fully understand physical requirements of ice hockey match-play.

Irrespective of the playing position, ice hockey players require adequate fitness to cope with the aforementioned demands of the game. Beyond the limited information available about ice hockey athletes’ characteristics using specific on-ice testing approaches (Vigh-Larsen et al., 2019, 2020b, 2020c), it is important to understand the practical relevance of these assessments by quantifying the relationship with match performance (e.g., ecological validity). However, to date only one study has attempted to quantify the associations between test performance and game activity variables in professional ice hockey players using sport-specific assessments (Lignell et al., 2018) and no information exists regarding match performance in relation to anaerobic-based on-ice tests (e.g., sprint and RSA) which might be relevant in relation to the imposed demands of a professional ice hockey game (Lignell et al., 2018; Montgomery, 1988; Rago et al., 2020, 2021; Vigh-Larsen et al., 2020a).

In summary, little evidence exists regarding the physical and physiological demands of professional ice hockey match-play with special emphasis on position-specific acceleration and deceleration profiles and potential fatigue development over the course of a game (Allard et al., 2020; Brocherie et al., 2018; Lignell et al., 2018; Stanula et al., 2016; Vigh-Larsen et al., 2020a). Thus, the main objective of this study was to describe the specific game activity profile of a professional ice hockey team with special emphasis on fatigue development and playing position. Specifically, the aims of this study were (i) to compare game activity profiles considering the game period and the playing position, (ii) to quantify the correlation between game activity variables, and (iii) to quantify the correlation between game activity variables and physical fitness.

## Methods

### Participants

Anthropometric characteristics, physical capacities and match activities were collected from 17 professional ice hockey players (6 defenseman and 11 forwards) competing in the highest Danish tier (Metal Liga). A detailed description of participant characteristics is reported in Table 1. Data were collected without interfering with regular training as established by the technical staff and all procedures followed the guidelines of the Declaration of Helsinki and were in line with established ethical standards (Winter and Maughan, 2009). All participants gave informed consent before initiating the experiment.

### Measures

**Table 1:** Anthropometric characteristics and physical capacities of elite Danish ice hockey players (n = 17).

Variable	Mean ± *SD*	Range
Age (yrs)	26.2 ± 4.7	18.0–34.0
Height (m)	181.1 ± 5.9	171.0–191.5
Body mass (kg)	81.6 ± 6.91	70.8–94.4
Muscle mass (%)	50.2 ± 2.0	47.5–55.6
Body fat (%)	13.6 ± 2.2	9.5–16.9
HR_max_ (bpm)	192.81 ± 7.04	183.00–210.00
10-m sprint (s)	1.74 ± 0.07	1.57–1.84
30-m sprint (s)	4.11 ± 0.11	3.95–4.31
RSA (s)	4.19 ± 0.10	4.00–4.34
Yo-Yo IR1-IH_SUB_ (%HR_max_)	85.29± 4.18	78.53–90.77

HR_max_, maximum heart rate, RSA, repeated sprint ability; Yo-Yo IR1-IH_SUB_, Submaximal Yo-Yo Intermittent recovery test ice hockey – level 1.

Game data were collected using a wearable accelerometric system and HR monitors (Polar Team Pro system 2, Polar, Kempele, Finland) throughout a four-week competitive period, between February and March 2020. Pre-game warm-up data were excluded. Only players who spent a minimum of 4 min on the ice were considered as criteria to normalize game data. No input was given about the research design to the technical staff throughout the data collection period. Data were collected in the context of the regular player’ monitoring routine. A total of 122 individual files from 8 competitive professional games (median [range], 8 [4–8] matches per player) were analysed.

Sprint performance (10- and 30-m), RSA and HR during the submaximal Yo-Yo Intermittent recovery ice hockey – level 1 test (Yo-Yo IR1-IH_SUB_) were assessed twice 30 days apart. The best results of these two evaluations (lowest sprint time and lowest HR) were retained as representative of the individual players. To remove the effect of game duration, correlations between physical capacity and game activity variables, as well as between game exposure and accelerometer- and HR-based variables were computed on individual time on ice.

### Design and procedures

#### Testing

The RSA test consisted of 3 × 30-m all-out efforts, interspersed by 25-s active recovery skating slowly back to the start. Each player initiated the sprint 0.5 m behind the first timing gates (Witty Gate Wireless Training Timer Photocells, Microgate, Italy, with a precision of 0.001 s) and a dual beam setup was used to prevent interference from swinging limbs or the stick accidentally activating the gates prematurely. Verbal encouragement was provided to motivate players to provide maximal effort each time. The mean time of the three sprints was recorded as an indicator of RSA, as previously described (Rampinini et al., 2007). The best (lower time) of these three sprints was also retained as the indicator of linear sprint ability using 10- and 30-m split times (intraclass correlation coefficient = 0.92 (Hajek et al., 2020)).

A 6-min submaximal version of the Yo-Yo IR1-IH_SUB_ with HR measurements (Polar Team Pro system 2, Polar, Kempele, Finland), as previously described (Lignell et al., 2018) was performed on the ice in the official ice rink of the team. The test was performed at 11.00 AM as the first part of a training session, 4 days after a game. The test consisted of 2 × 20-m shuttle skates with gradual speed increments signalled by audio beeps, interspersed by a 10-s passive recovery period. The mean HR during the last 30 s was retained as an indicator of cardiorespiratory fitness (Buchheit, 2014; Krustrup et al., 2003; Rago et al., 2019), and was expressed as a percentage of the individual HR_max_ (obtained in a previously-described ice-hockey specific incremental test (Stanula et al., 2016)). HR responses during Yo-Yo IR1-IH _SUB_ have been reported to present good criterion validity (very large correlation with directly-measured maximal oxygen uptake; *r* = - 0.85) and ecological validity (moderate correlation with game performance; *r* = -0.55 to -0.47) in ice hockey players (Lignell et al., 2018).

#### Match analysis

Acceleration and HR data were recorded using a wearable device incorporating a 200-Hz accelerometer and a gyroscope (Polar Team Pro system, Polar, Kempele, Finland) and a 1-s interval telemetric system, respectively. The device (weight: 39 g; dimension: 36 × 68 × 13 mm) was placed on the lower sternum using an elastic band. Data were stored in the device and downloaded using the manufacturer’s software (POLAR Team Pro, Software version 1.3.1, POLAR, Polar Electro Oy, Kempele, Finland). Acceleration variables included the number of total accelerations (Acc_tot_), accelerations above 2 m·s^-2^ (Acc2; as criteria of an intense increase in speed), total decelerations (Dec_tot_) and decelerations below -2 m·s^-2^ (Dec2; as criteria of an intense decrease in speed). These were expressed as absolute values and relative to on-ice game exposure.

The physiological demands of the game were expressed as mean HR (HR_mean_), peak HR (HR_peak_) during exercise (including both shifts and time on the bench, and excluding breaks between periods) and time spent above 85% HR_max_ (t85HR) based on a recent ice-hockey study (Vigh-Larsen et al., 2020a).

### Statistical analyses

The Kolmogorov-Smirnov test revealed that game data and physical capacity data were normally distributed (*p* > 0.05). Differences between periods of the match and playing positions were analysed using a mixed model taking into account that different players took part in a different number of matches (Cnaan et al., 1997). The periods of the game and playing positions were set as a fixed effect, the individual player was set as a random effect, and acceleration and HR data were set as dependent variables. When a significant effect was found, pairwise comparisons were carried out using a Bonferroni post-hoc test. To describe the magnitude of differences, the t statistics derived from the mixed model were converted to effect sizes’ correlations (*r*) (Rosnow et al., 2000). Within-player correlations between match activity variables were calculated to take into account the repeated-measure nature of this study (Bland and Altman, 1995). Additionally, correlations between physical capacity and game demands were computed using paired-sample correlations on the mean within-player game values across different games. The magnitude of correlations was interpreted using the following criteria: *r* ≤ 0.1 (trivial), 0.1–0.3 (small), 0.3–0.5 (moderate), 0.5–0.7 (large), 0.7–0.9 (very large) and ≥0.9 (almost perfect).

Statistical significance was set at *p* ≤ 0.05. Data analyses were performed using Statistical Package for Social Science software (IBM SPSS Statistics for Windows, Version 25.0. Armonk, NY).

## Results

### Game demands by game periods and playing positions

**Figure 1:**
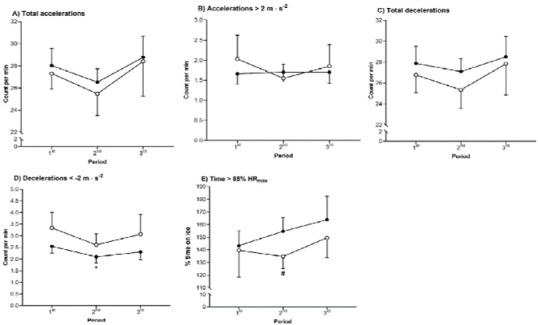
Match activity profile of an elite male ice hockey team expressed as relative values to time on ice. Data are estimated marginal means and 95% confidence intervals; the white symbols are defensemen and the black symbols are forwards (circles relative to total duration and triangles relative to time on ice); HR_max_, maximum heart rate; * compared to the 1^st^ period, and # compared to defenders.

Irrespective of the playing position, the game duration was largely longer in the 3^rd^ period compared to the 1^st^ and the 2^nd^ period (*r* = 0.56– 0.59; *p* < 0.01; Table 2). Forwards performed a significantly higher number of accelerations in the 2^nd^ period compared to the 1^st^ period (*r* [95%CIs] = 0.56 [0.29–0.75] *p* = 0.001; Table 2). When game data were expressed in relation to time on ice, forwards experienced moderate declines in Dec2/min during the 2^nd^ period (*r* [95%CI] = 0.36 [0.06–0.60]; *p* = 0.042; Figure 1D).

**Table 2:** Overall game demands in an elite Danish ice hockey team (n = 122 files).

Variable	Playing position	1^st^ period	2^nd^ period	3^rd^ period
Total duration (min)	Defensemen	31.61 ± 0.47	31.54 ± 1.64	36.78 ± 5.47 ^a,b^
	Forwards	31.61 ± 0.46	31.50 ± 1.61	37.33 ± 5.54 ^a,b^
Time on ice (min)	Defensemen	6.56 ± 1.14	6.46 ± 1.68	6.65 ± 1.82
	Forwards	5.09 ± 1.24 *	4.50 ± 1.32*	4.85 ± 1.57 *
Total Acc (count)	Defensemen	177.00 ± 20.00	165.15 ± 22.48	181.62 ± 30.14
	Forwards	138.82 ± 27.58 *	118.83 ± 32.57 *	137.89 ± 42.22 *
Acc > 2 m·s^-2^ (count)	Defensemen	12.36 ± 4.94	10.00 ± 4.22	11.54 ± 4.81
	Forwards	8.57 ± 3.99 *	7.28 ± 3.17 *	8.00 ± 3.43 *
Total Dec (count)	Defensemen	172.79 ± 19.23	164.46 ± 24.98	178.85 ± 32.34
	Forwards	137.89 ± 26.63 *	120.93 ± 31.17 *	136.54 ± 41.06 *
Dec < -2 m·s^-2^ (count)	Defensemen	21.43 ± 5.85	17.23 ± 5.34	19.62 ± 8.33
	Forwards	12.96 ± 4.58 *	8.97 ± 4.5 *,^a^	11.46 ± 5.53 *
Time > 85% HR_max_ (min)	Defensemen	8.49 ± 4.85	8.63 ± 5.6	9.73 ± 5.67
	Forwards	7.03 ± 4.24 *	6.80 ± 4.35 *	7.58 ± 4.70 *
HR mean (% HR_max_)	Defensemen	74.81 ± 3.87	75.27 ± 3.67	75.14 ± 3.67
	Forwards	71.29 ± 3.96 *	71.91 ± 4.89 *	71.68 ± 6.60
HR peak (% HR_max_)	Defensemen	98.97 ± 5.71	98.15 ± 1.45	97.43 ± 0.77
	Forwards	97.05 ± 1.11	97.92 ± 1.57	97.66 ± 1.54

Acc, accelerations; Dec, decelerations; HR, heart rate; HR_max_, maximum heart rate; * denotes significant differences compared to defenders, ^a^ to the 1^st^ period and ^b^ to the 2^nd^ period (p < 0.05).

Irrespective of the period of the game, forwards performed a moderately higher number of Acc_tot_, Acc2, Dec_tot_, Dec2 and spent more t85HR than defenders (*r* = 0.33–57; *p* < 0.01; Table 2). When expressed as relative to individual time on ice, defenders performed a greater number of Dec2/min than forwards in all periods (*r* = 0.46– 0.57; *p* < 0.05; Figure 1D). However, forwards spent more percentage of t85HR than defenders during the 2^nd^ period (*r* = 0.32 [0.01–0.58]; *p* = 0.037; Figure 1E).

### Correlations between game activity variables

**Figure 2:**
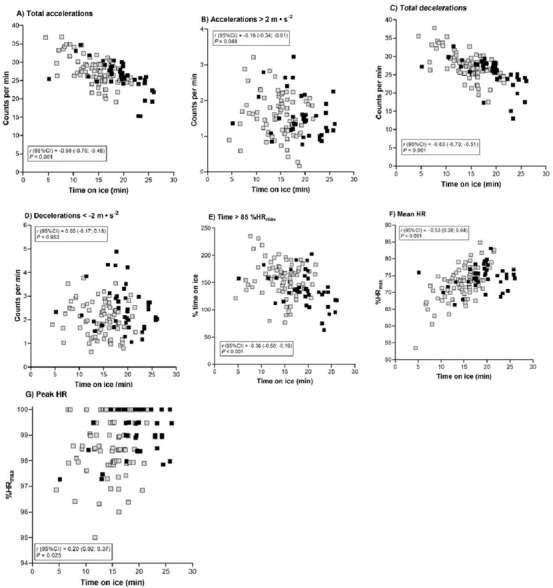
Correlations between time on ice and activity demands during elite ice hockey games (n = 122). The black dots are defensemen; the grey dots are forwards; HR, heart rate; HR_max_, maximum heart rate.

The time spent on ice was inversely correlated with Acc_tot_/min, Acc2/min, Dec_tot_/min, Dec2/min and the percentage of t85HR (*r*= -0.63 to -0.18; *p* < 0.05) and positively correlated with HR_mean_ and HR_peak_ (*r* [95% CI] = 0.53 [0.38–0.64] and 0.20 [0.02–0.37]; *p* < 0.05; Figure 2).

In absolute values, Acc_tot_ was positively correlated to Acc2, Dec_tot_, Dec2 and t85HR (*r* = 0.55–0.96; *p* < 0.01). Moreover, Acc2 was moderately to largely correlated to Dec_tot_, Dec2 and t85HR (*r* = 0.43–0.77; *p* < 0.01). When expressed as relative values to time on ice, positive correlations were observed between Acc_tot_/min and Dec_tot_/min and between Acc2/min and Dec2/min (*r* [95%CI]= 0.86 [0.65; 0.95] and 0.69 [0.31; 0.88], respectively; *p* < 0.01). A detailed description of the within-subject correlations between game activity variables is reported in Table 3.

**Table 3:** Within-subjects correlations between game activity variables (n = 122 files). Data are presented as magnitude of correlations with 95% confidence intervals.

		Total Acc	Acc > 2 m·s^-2^	Total Dec	Dec < -2 m·s^-2^
*Absolute values*					
Acc > 2 m·s^-2^	*r*	0.55 (0.10; 0.82)	-	-	-
	*p*	0.002	-	-	-
Total Dec	*r*	0.96 (0.90; 0.99)	0.58 (0.14; 0.83)	-	-
	*p*	<0.001	0.001	-	-
Dec < -2 m · s^-2^	*r*	0.61 (0.18; 0.84)	0.73 (0.39; 0.90)	0.60 (0.16; 0.84)	-
	*p*	<0.001	<0.001	<0.001	-
Time > 85%	*r*	0.76 (0.44; 0.91)	0.43 (-0.06; 0.76)	0.77 (0.46; 0.91)	0.40 (-0.10; 0.74)
HR_max_	*p*	<0.001	0.054	<0.001	0.061
*Relative to time on ice*					
Acc > 2 m · s^-2^	*r*	0.33 (-0.18; 0.70)	-	-	-
	*p*	0.149	-	-	-
Total Dec	*r*	0.86 (0.65; 0.95)	0.37 (-0.13; 0.72)	-	-
	*p*	<0.001	0.087	-	-
Dec < -2 m · s^-2^	*r*	0.37 (-0.13; 0.72)	0.69 (0.31; 0.88)	0.38 (-0.13; 0.73)	-
	*p*	0.873	<0.001	0.082	-
Time > 85%	*r*	0.44 (-0.05; 0.76)	0.25 (-0.26; 0.65)	0.38 (-0.12; 0.73)	0.19 (-0.32; 0.61)
HR_max_	*p*	0.062	0.285	0.079	0.431

Acc, accelerelations; Dec, decelerations; HR_max_, maximum heart rate.

No significant correlations were found between individual characteristics and game activity variables scaled by individual time on ice (*p* > 0.05).

## Discussion

The present study provides new knowledge on physical and physiological demands of professional ice hockey match-play with special emphasis on position-specific acceleration and HR profile. The main findings of this study are that (i) no clear signs of fatigue appear towards the end of a game; (ii) defensemen appear to experience greater physical and physiological demands than forwards, with similar demands in relation to individual time spent on ice; (iii) an inverse relationship exists between time on-ice and the total number of accelerative efforts; and (iv) anthropometric characteristics and skating-based physical capacity are not associated to the work rate during official professional ice hockey matchplay.

In contrast to previous studies, we did not observe a significant decrease in the number of Acc_tot_ or Acc2 and Dec2 during the 3^rd^ period, neither when expressed in absolute nor relative terms to on-ice exposure (Allard et al., 2020; Lignell et al., 2018; Vigh-Larsen et al., 2020a). However, we observed a decrease in the absolute and relative amount of Dec2 in forwards during the 2^nd^ period, but this was preserved during the 3^rd^ period. However, the 3^rd^ period was characterised by a longer duration, possibly due to more or longer game interruptions (play stoppages, time out usage, etc.), enabling players to recover between intense efforts and maintain performance. Accordingly, an inverse relationship between total accelerations and decelerations and individual time on ice was observed in the present study, revealing that the most frequently used players performed the lowest amount of changes in speed and direction per minute over the course of the game likely due to pacing strategies in order to avoid exhaustion prematurely or due to shorter rest between each shift throughout the game compromising performance.

Thus, the combination of extended recovery periods due to more interruptions and possible application of pacing strategies for the most frequently used players may have minimized fatigue development toward the end of the game. Also, a decrease in performance cannot be ruled out for specific players, but our sample size did not allow for accurate sub-analyses of match-activities grouped according to playing exposure. Potential fatigue development during a game could be related to fatiguing mechanisms associated with a marked degradation in muscle glycogen (~53%) with a major part of both type I and II fibres being depleted towards the end of an ice hockey game (Vigh-Larsen et al., 2020a) as well as significant hyperthermia and associated dehydration (Logan-Sprenger et al., 2011).

No between-period differences were observed in HR-based variables, which is in accordance with previous findings (Stanula et al., 2016; Stanula et al., 2014). However, our work is in contrast to that of Vigh-Larsen et al. (2020a) which reported a decreased HRmean in the 3rd period, compared to the 1st and the 2nd period, with a concurrent reduced time in high HR zones during the 3rd period in an experimental game. Our findings could possibly be explained by the frequency of substitutions performed by the team, which may have counteracted an increased HR toward the end of the game. A further reason could be the fact that the aforementioned study was conducted using an experimental game with all players having a uniform time on ice, which reflects game demands of the most frequently used players only (Vigh-Larsen et al., 2020a). Furthermore, beyond playing position, we did not account for several game-related contextual variables. Indeed, a recent match-analysis report using 4886 offensive actions from 192 Swedish top-tier ice hockey games revealed that numbers of involved players and the specific game periods significantly affected goal-scoring opportunities (Lignell et al., 2020). However, no information is available regarding how game-related situational variables (f. ex. playing position, the number of players involved, match period, game location and opponent standard) affect physical and physiological demands of an ice hockey game. It is important to note that development of fatigue is difficult to be observed when considering HR variables in ice hockey as the average shift duration is about 40 s, with players exercising at supramaximal intensities (Montgomery, 1988). Thus, the aerobic mechanisms (associated to HR) leading to fatigue variables could have been skewed.

In the present study, defensemen experienced greater absolute physical and physiological demands than forwards, whereas loading relative to time on ice was similar. In absolute terms, irrespective of the game period, defenders performed a moderately higher number of Acctot, Acc2, Dectot, Dec2 and spent more t85HR than forwards (r = 0.33–0.57). Our results contrast previous findings from Allard et al. (2020) demonstrating similar total loads across playing positions and less on-ice loads per min for defensemen. However, based on the correlation between speed, acceleration and HR variables in team sports games (McLaren et al., 2018), the present results are partially supported by those of Lignell et al. (2018) showing greater moderate (14–17 km·h^-1^) and fast (17–21 km·h^-1^) skating speed in defensemen during one official game. On the other hand, the same authors reported greater very fast (21–24 km·h^-1^) and sprint (>24 km·h^-1^) skating distance covered by forwards in absolute numbers and substantially larger amounts of high-intensity skating relative to on-ice duration (Lignell et al., 2018). Similarly, Douglas and Kennedy (2020) reported significantly more fast, very fast and sprint skating in forward players, but more slow and moderate speed skating in defensemen. This inconsistency could be due to different positional activity profiles with forwards performing more long fast-paced breaks from end-to-end producing high amounts of high-speed skating distance. In contrast, defenders may cover more ground in the area in front of the goal with frequent small moves from side to side producing a higher number of accelerations and decelerations, but less high-speed skating. This might, at least partially, suggest that acceleration and deceleration work possibly underestimate the demands for forwards whereas speed- or distance-based metrics likely underestimates the demands for defensemen. Additionally, we provided only total time on ice per period (not per shift) to compare activity demands by playing position, which automatically results in longer effective playing time on ice for players of defensive formations. Thus, future studies should include both distance- and accelerometer-based metrics, as well as consider shift duration to fully comprehend positional requirements.

Our within-subject correlation analysis revealed that the time spent on ice was inversely correlated with Acctot, Acc2, Dectot, Dec2 and t85HR. These negative associations may suggest that players could have adopted pacing strategies to manage fatigue, due to game-related situational factors (Waldron and Highton, 2014). However, the positive correlations between HR_mean_ and HR_peak_ may indicate an increased aerobic contribution with game exposure (Mann et al., 2013). Most game associations between activity variables were present when match data were not adjusted for individual time on ice. Despite the different variables, our positive relationships observed in Acctot and Dectot as measures of exercise volume with Acc2, Dec2 and t85HR as load measures are partially supported by Lignell et al. (2018) reporting a positive correlation between total distance and high-intensity distance during one official ice hockey game. Additionally, the positive relationship between Acc2, Dec2 and t85HR is supported by other team sports during official competitions or drills (McLaren et al., 2018). On the other hand, when game data were adjusted to individual time on ice, positive correlations were found only between Acctot/min and Dec/min and between Acc2/min and Dec2/min. This may indicate that players who perform more accelerative efforts at a given intensity, also brake often at a similar negative intensity. Our findings are in contrast to those of several team sports reporting consistent associations between external (e.g. speed, accelerations) and internal (e.g. HR, perception of effort) load metrics (McLaren et al., 2018).

This is the second study to examine game activity variables in relation to individual physical characteristics in male professional ice hockey (Lignell et al., 2018). In the present study, anthropometric characteristic and skating-based physical capacity were not associated to the work rate (adjusted to individual time on-ice). This could be partially due to the fact that better-conditioned players were likely playing for longer shifts during the matches and therefore had to slightly manage their work rate. Our findings are in contrast to recent findings in 245 ice hockey players of different competitive standards, showing that quickest players over 33.15 m and the most agile players have low body fat content and greater muscle mass, although these relationships were predominantly weak (Vigh-Larsen et al., 2019). While accounting for the different match demands based on continuous time and running instead of skating, our findings are also in contrast to those observed in professional soccer games, showing a negative association (r = -0.57) between the players’ percentage of fat mass and the number of sprints performed during official soccer games (Radzimiński et al., 2020; Rampinini et al., 2007; Redkva et al., 2018). Additionally, lack of a relationship between RSA and match performance in our study is in contrast to findings of Rampinini et al. (2007) who examined Italian Premier league soccer and showed that RSA mean was inversely correlated to distance covered above 19.8 and 25.2 km·h^-1^ (r = -0.65 to -0.60). Similarly, our lack of association between HR responses to the Yo-Yo IR1-IHSUB is in contrast to Lignell et al. (2018) who showed negative associations with high-intensity skating distance and the number of intense bouts (r = -0.85 to - 0.54). However, the latter is in partial agreement with a recent study in Spanish soccer players suggesting the relevance of this test based on a moderate correlation (r = -0.56) with total distance covered, although no correlation was observed with sprinting distance (Rago et al., 2019). Acknowledging the lack of information on the fitness determinants of game performance in ice hockey, it is difficult to speculate about our results. While accounting for the different sports demands, the lack of significant relationships could be partially explained by the time-motion variables and the equipment adopted in soccer. For example, these studies used speed-based variables (Rago et al., 2019; Rampinini et al., 2007) which might possibly underestimate the energetic cost associated with acceleration/deceleration running (Akenhead et al., 2016). Furthermore, the aforementioned studies used multiple-camera computerized tracking systems which might present a different systematic error compared to wearable technology such as the global positioning system, local positioning system or accelerometric systems (Buchheit et al., 2014; Randers et al., 2010). Further research using a wide range of on-ice fitness tests (Roczniok et al., 2015; Vigh-Larsen et al., 2019, 2020b, 2020c) is warranted to clarify the relationship between physical capacity and game performance in ice hockey.

Despite analyses of multiple matches, our sample size is limited to one team, and thus, the observed differences in physical and physiological demands between defensemen and forwards could be partially explained by game-or-team specific situational factors (Lignell et al., 2020) or strategies adopted by the coach. This also clearly affects the examination of fatigue, which was performed using indirect measurements of fatigue (changes in activity patterns) without any objective pre- and post-match assessments. Secondly, we only reported accelerations and decelerations in our movement analysis and not the distance covered in different speed zones as in previously studies (Lignell et al., 2018; Vigh-Larsen et al., 2020a). Therefore, it is difficult to compare our findings with existent ice hockey evidence.

In summary, the present study characterized the physical and physiological demands of ice hockey matches with special emphasis on fatigue patterns and playing position, as well as correlations of match activity variables, using a set of multiple games. This study provides further insights into performance and physiology of ice hockey, being of potential interest for coaches and any professionals involved with ice hockey players.

Defensemen experienced greater absolute physical demands than forwards, but similar demands when acceleration and HR data were expressed in relation to time on ice. No clear signs of fatigue were captured as indicated by stable physical performance between game periods. However, players who spent more effective playing time on ice performed less accelerations and decelerations, possibly indicating compromised physical performance due to reduced rest periods on the bench, or possible pacing strategies to manage fatigue. Additionally, individual anthropometric characteristics and skating-based physical capacities seem not to be representative of game-related performance, possibly indicating that well-conditioned players were employed for longer shifts.
